# Ratio of C-Reactive Protein/Albumin is An Inflammatory Prognostic Score for Predicting Overall Survival of Patients with Small-cell Lung Cancer

**DOI:** 10.1038/srep10481

**Published:** 2015-06-18

**Authors:** Ting Zhou, Jianhua Zhan, Shaodong Hong, Zhihuang Hu, Wenfeng Fang, Tao Qin, Yuxiang Ma, Yunpeng Yang, Xiaobo He, Yuanyuan Zhao, Yan Huang, Hongyun Zhao, Li Zhang

**Affiliations:** 1 Department of Medical Oncology, Sun Yat-sen University Cancer Center, Guangzhou, China; 2 State Key Laboratory of Oncology in South China, Guangzhou, China; 3 Collaborative innovation Center for Cancer Medicine, Guangzhou, China; 4 Department of Oncology, The Fifth Affiliated Hospital of Sun Yat-sen University, Zhuhai, China

## Abstract

Recent studies have indicated that the C-reactive protein/ albumin (CRP/Alb) ratio is associated with clinical outcomes in patients with hepatocellular carcinoma (HCC). We examined the prognostic value of this ratio in patients with small-cell lung cancer (SCLC). In this retrospective study, a total of 367 eligible SCLC patients were analyzed and the correlation between the pretreatment CRP/Alb ratio and overall survival (OS) was investigated. The optimal cutoff level of CRP/Alb ratio was at 0.441. A low and high CRP/Alb ratio was assigned to 65.1% and 34.9% of patients, respectively. The median OS of patients with a high CRP/Alb ratio was worse than those in the low group (13.70 vs 18.90 months HR, 1.34; p = 0.005). Disease stage (p < 0.001), performance status (PS) (p < 0.001) and pretreatment LDH (p < 0.001) were also significant predictors of OS. Multivariate analyses showed that the CRP/Alb ratio is an independent prognostic factor (p = 0.025). This study demonstrated that the CRP/Alb ratio could independently predict OS in patients with SCLC, and had comparable prognostic value to other known prognostic markers. Therefore, the CRP/Alb ratio could have prognostic value and be a measurable biomarker in patients with SCLC.

Lung cancer is a commonly diagnosed cancer and is the leading cause of cancer-related deaths. In 2014, approximately 224,210 new lung cancer cases are predicted and 159,260 will die from this malignancy in the United States^1^. Of the lung cancers diagnosed, 15% are classified as small-cell lung cancer (SCLC)^2^,^3^. Staging of patients with SCLC is determined by the Veteran Affairs Lung Study Group (VALSG) staging system^4^. First-line treatment using etoposide-based chemotherapy produces a high response rate in patients with SCLC. However, the 5-year overall survival rate for patients with limited and extensive staging is 25% and 7.8%, respectively^5^,^6^. Currently, although thoracic radiotherapy and prophylactic cranial irradiation have improved the clinical outcome of patients with SCLC, a proportion of patients will develop relapse and/or distant metastases^7^. These patients have a poorer prognosis. Therefore, identifying factors with prognostic value in patients currently treated for SCLC may help improve their clinical outcomes.

Various laboratory and clinical markers include baseline serum carcinoembryonic antigen (CEA), lactate dehydrogenase (LDH), neuron-specific enolase (NSE), performance status (PS), disease extent, age, and gender. These prognostic markers are associated with overall survival (OS) in SCLC^8^,^9^,^10^. Of the prognostic factors, PS and disease stage are important prognostic indicators for SCLC^11^,^12^. However, conflicting results of these prognostic factors have been reported and there remains no optimal prognostic index for SCLC.

Although environmental and genetic factors contribute to the development of cancer, there is increasing evidence which demonstrates the role of inflammation in the initiation and progression of cancer^13^. The prognostic value of many inflammation–based scores, such as the modified Glasgow Prognostic Score (mGPS), prognostic Nutritional Index (PNI), and neutrophil Lymphocyte Ratio (NLR), have been validated in many types of cancer, including SCLC^14^,^15^,^16^. Additionally, several studies have indicated that the levels of C-reactive protein (CRP) correlates with the disease progression in a number of cancers. It has been reported that serum albumin could be a marker of tumor recurrence^17^,^18^,^19^. The prognostic value of CRP and serum albumin is unknown and may have independent prognostic value. A previous study showed that CRP/Alb ratio is an independent risk factor of mortality in patients with sepsis^20^. Moreover, it has been shown that the C-reactive protein/ Albumin (CRP/Alb) ratio is significantly associated with the outcome of patients with cancer^21^.

In this retrospective study, we examine the prognostic value of CRP/Alb ratio in patients with SCLC. Additionally, we further investigate the relationship between CRP/Alb ratio and clinical outcomes.

## Materials and Methods

### Participant Identification

We enrolled 665 patients diagnosed with SCLC in Sun Yat-sen University Cancer Center (SYSUCC) between January 2006 and December 2011. The inclusion criteria of this study were as follow: 1) cytologically or histologically diagnosed as SCLC, 2) an age of at least 18 years, 3) having at least one measurable lesion, 4) having pre-treatment blood sampling for CRP and albumin measurement, and 5) staged on the basis of VALSG staging system. Patients were excluded if there is detectable inflammatory disease. Moreover, the propensity score is used to adjust for potential selection bias of the participants. Then a total of 367 eligible patients were enrolled into the study. All clinical and follow-up records were reviewed retrospectively. This study was approved by the Institutional Review Board of SYSUCC and written informed consent was obtained for each patient.

### Clinical Data extraction

We collected baseline characteristic of participants, including age, gender, disease stage, PS, smoking status, and histology by using a standard data extraction system. Patients who had more than 100 cigarettes were defined as smokers. Stage was determined based on the VALSG staging system, which divides patients into limited and extensive stage. Blood samples were tested prior to initial treatment for levels of CRP, albumin, and LDH. The CRP/Alb ratio was calculated by dividing the serum CRP level by the serum albumin level^20^,^22^.

### Patients Follow-up

All patients were carefully followed after pathological diagnosis. Patients received dynamic computed tomography (CT) scan every 2 cycles of chemotherapy or every 6 weeks. The response of treatment was evaluated by a systematical radiologic review committee according to the Response Evaluation Criteria in Solid Tumors (RECIST 1.0). We compared the difference in survival by means of OS, defined as the time elapsed from the date of pathological diagnosis to the time of death for any cause or at last follow-up. The initiation of follow-up was the date of finishing the anti-tumor treatment, and the end date of follow-up was March 30, 2014 or death from any cause. Patients who did not die at the time of last follow-up were censored.

### Statistical Analysis

Continuous variables were presented as median value and range, and were transformed into dichotomous variables at median value. The Chi-Square or Fisher’s exact test was used to compare the Categorical variables, which was presented as the numbers and percentages of patients. The optimal cutoff level of CRP/Alb ratio was determined by a web-based system, R software-engineered, designed by Budczies J *et al.* ( http://molpath.charite.de/cutoff/)^23^. The relationship between OS and prognostic factors, which includes cancer stage, LDH, CRP/Alb ratio and PS, was calculated using the Kaplan–Meier method respectively. Univariate analyses were conducted using the log-rank test analysis. Multivariate analysis of these variables in survival was performed using the Cox proportional hazards model. All statistical analyses were performed using SPSS 21.0 software (IBM, Armonk, NY). All tests were two-sided and a P value < 0.05 was considered statistically significant.

## Results

A total of 367 enrolled patients were diagnosed with SCLC. Pre-treatment evaluation of CRP and albumin was performed between January 2006 and December 2011. The assessment of PS and disease stage was taken at time of patient admission. The majority of the patients enrolled were males (*n* = 316, 86.1%) and smokers (*n* = 302, 82.3%), and had a PS of 0-1 (*n* = 337, 91.8%). The median age was 59 years (range: 23-82 years). Among these, 180 (49.0%) patients were in limited stage and 187 (51.0%) patients were in extensive stage. Most of the enrolled patients received etoposide-based chemotherapy as first-line treatment (*n* = 315, 85.8%), while 81 (22.1%) patients had prophylactic cranial irradiation (PCI) after chemotherapy. The baseline characteristics of the 367 patients are shown in Table 1.

**Figure 1:**
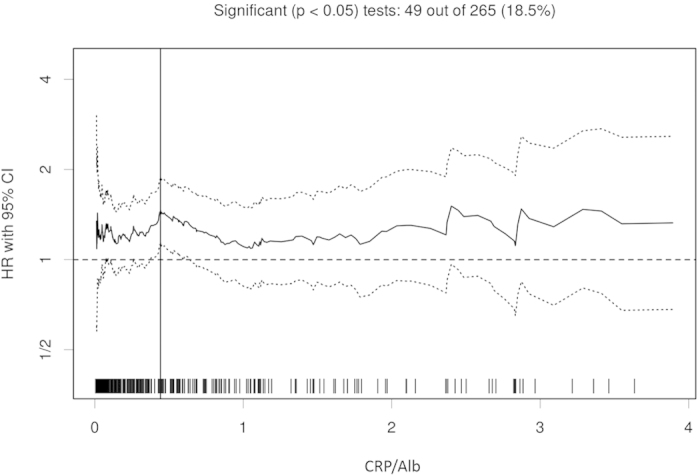
Hazard ratio (HR) for overall survival (OS) independent of cutoff point for CRP/Alb ratio in patients with small-cell lung cancer. The vertical line designates the optimal cutoff point with the most significant (log-rank test) split. The plots were generated using Cutoff Finder.

Using the biostatistical tool, Cutoff Finder, we found that the range of cutoff value for CRP/Alb ratio was wide and determined 0.441 as the optimal cutoff level for assessing OS^23^ (Fig. 1). Patients were divided into two groups based on the cutoff value of CRP/Alb ratio ≥ 0.441 (n = 128, 34.9%) and CRP/Alb ratio <0.441 (n = 239, 65.1%).

The clinicopathological characteristics of patients based on CRP/Alb ratio are described in Table 2. An elevated CRP/Alb ratio was significantly associated with the abnormally higher LDH level (*p* = 0.010). However, compared to the high CRP/Alb ratio group, gender (*p = *0.637), age (*p = *0.659), PS (*p = *0.734), chemotherapy regimen (*p = *0.432) and smoking status (*p = *0.669) of patients were similar in the low CRP/Alb ratio group.

**Figure 2:**
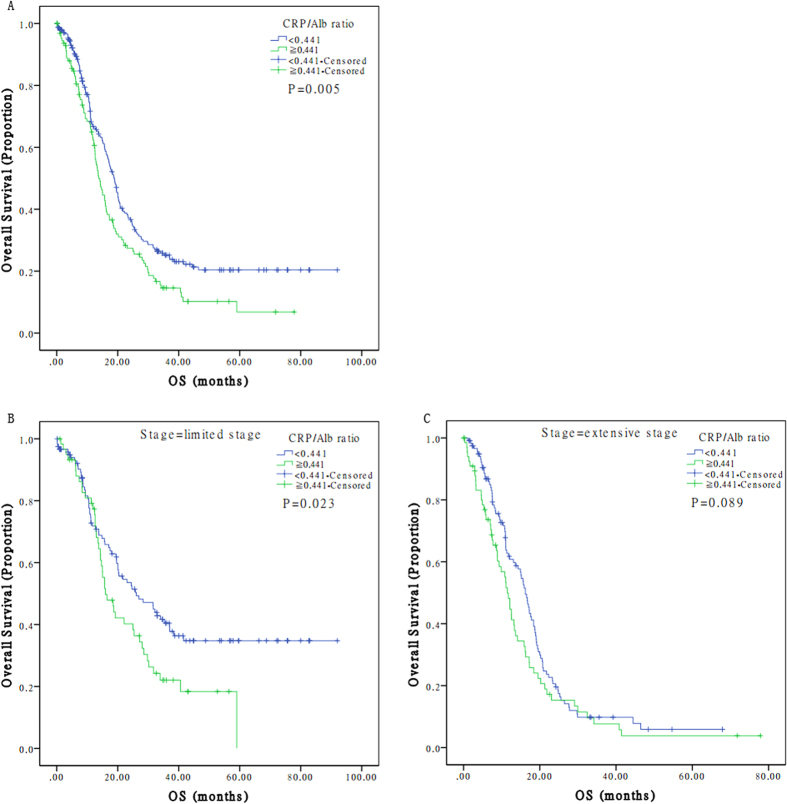
The prognostic value of CRP/Alb ratio on overall survival curves in patients with limited or extensive stage disease. **A**. Comparison of OS on patients with high CRP/Alb ratio *vs* low CRP/Alb ratio. **B**. Comparison of OS on patients in limited stage with high CRP/Alb *vs* low CRP/Alb ratio. **C**. Comparison of OS on patients in extensive stage with high CRP/Alb *vs* low CRP/Alb ratio

A total of 128 (34.9%) patients were categorized into the high level group of CRP/Alb ratio, while the remaining 239 (65.1%) patients were stratified into the low level group. In contrast to the patients with high CRP/Alb ratio, patients with low CRP/Alb ratio had longer overall survival (18.9 *vs* 13.7 months, *p* = 0.005) (Table 1). Similarly, longer overall survival was also observed when patients were stratified into limited stage (*p* = 0.023), but not extensive stage (*p* = 0.089) (Fig. 2A–C).

**Figure 3:**
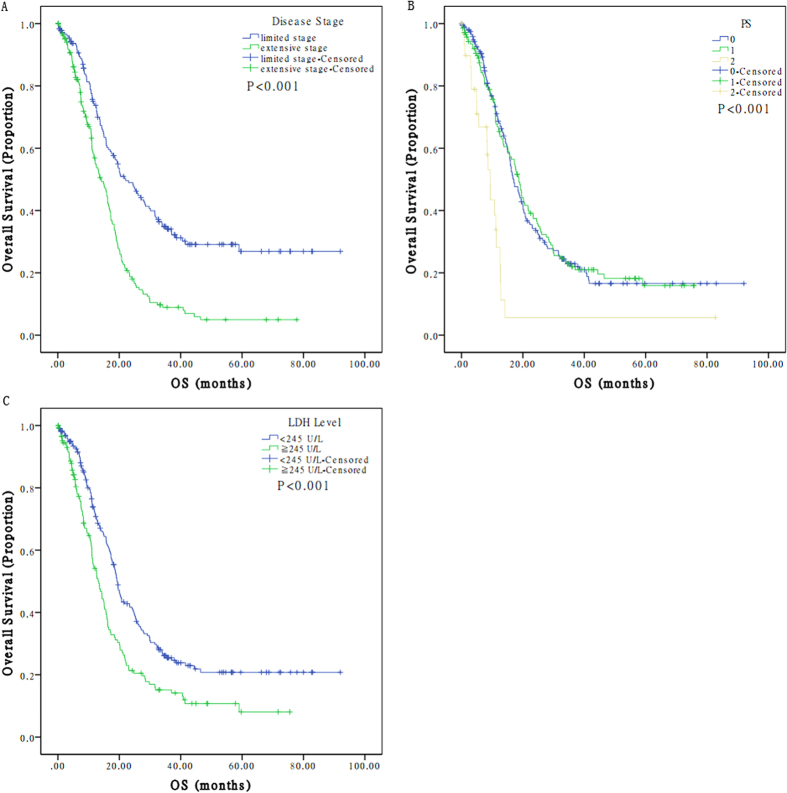
Overall survival curves comparing patients with **A**. limited disease vs extensive disease; **B**. good vs bad PS; **C**. high LDH vs low LDH

The median follow-up time was 29.40 months (range: 0.03-116.07 months). During the follow-up period, 258 (70.3%) patients died, and the ratio for loss to follow-up was 9.5% (*n* = 35). The median OS of the 367 eligible patients was 13.8 months (range: 0.03-92.03 months). Disease stage (*p* < 0.001), LDH level (*p* < 0.001), age (*p* = 0.049) and PS score (*p* < 0.001) were significantly associated with OS by univariate analyses (Fig. 3A–C). Furthermore, PS had distinct prognostic value in patients with limited (*p* = 0.024) or extensive staging (*p* < 0.001). However, when stratified by staging, a significant correlation between LDH level and OS was found in patients with extensive (P = 0.003), but not limited staging (*p* = 0.097). There were no significant association between OS and gender (*p* = 0.077), smoking status (*p* = 0.917), chemotherapy regimen (*p* = 0.685), and prophylactic cranial irradiation (*p* = 0.361) (Table 1).

Using multivariate analyses, we then tested for correlation among the different variables. The analyses revealed that CRP/Alb ratio is an independent prognostic factor in patients with SCLC (*p* = 0.025). Compared to patients with a low CRP/Alb ratio ( < 0.441), those with high CRP/Alb ratio ( ≥ 0.441) were estimated to have 1.34 times higher risk of death (HR, 1.34; 95% CI, 1.04-1.73; *p* = 0.025). Moreover, cancer stage (*p* < 0.001), PS (*p* < 0.001) and LDH level (*p* = 0.008) also independently predicted OS (Table 3).

## Discussion

In this study, we retrospectively analyzed the prognostic power of CRP/Alb ratio in 367 eligible patients with SCLC at our cancer center. To our knowledge, this is the first study to analyze the correlation between CRP/Alb ratio and OS in patients with SCLC. The results of this demonstrated that CRP/Alb ratio is an independent prognostic indicator for patients with SCLC.

SCLC is an aggressive malignancy that is sensitive to cytotoxic agents and radiotherapy. However, most patients will die from the rapid growth of the cancer and acquired drug resistance associated with treatment^3^. Overall survival is an important index for evaluating clinical efficacy of different types of therapy. An inapposite predictor of OS may underestimate therapeutic benefits. Therefore, an accurate prognostic indicator to select those who are likely to benefit from anti-tumor treatment is needed.

Previous studies have reported that PS score, disease extent, LDH level, CEA and NSE significantly correlates with overall survival benefit in patients with SCLC^8^,^24^,^25^,^26^. These findings are consistent with the results of our studies, which demonstrated that better PS, limited staging, and normal serum levels of LDH were significantly associated with a longer survival than those with patients with a poor PS, extensive staging, and elevated serum LDH levels (Table 1). However, it remains uncertain whether these clinical variables have prognostic value in patients with SCLC. Furthermore, limitations in PS, heterogeneity of patients in the same disease stage, and uncertainties regarding increased LDH level may limit the prognostic value of these factors in predicting OS in SCLC.

Recent studies have showed a potential relationship between chronic inflammation and cancer. Inflammatory cells may alter the tumor microenvironment, which can promote tumorigenesis by increasing the proliferation, migration, and immune escape of tumor cells^27^. These observations suggest a correlation between chronic inflammation and poor OS in patients with cancer. Several studies have shown a link between chronic inflammation and tumorigenesis^28^,^29^. IL-6 is an important proinflammatory cytokine that plays a key role in the development of cancer and closely correlates with CRP levels. It has been reported that CRP is a sensitive and reliable prognostic marker for systemic inflammation that is also convenient for testing with standardized parameters established in clinical laboratories^30^,^31^. Hong *et al.* observed that high CRP level is associated with poor prognosis of patients with SCLC^32^. Moreover, serum albumin level was also a prognostic factor in SCLC patients. Low level of albumin is associated with malnutrition and weight loss, which results in a poor PS and increased cancer-related mortality^33^.

We hypothesized that merging CRP and albumin into a new index may have prognostic value in inflammation, and better predict overall survival of patients with cancer. Fairclough *et al.* proposed the concept of CRP/Alb ratio^22^. Otavio *et al.* indicated that CRP/Alb ratio showed predicted mortality in patients in ICU^20^. Furthermore, Akiyoshy *et al.* demonstrated that CRP/Alb ratio was a prognostic factor in patients with hepatocellular carcinoma (HCC). Therefore, we proposed that the CRP/Alb ratio could be a prognostic factor for patients with SCLC. In this study, a 0.441 cutoff value for CRP/Alb ratio was used for predicting overall survival in SCLC. In our study, a univariate analysis showed that the CRP/Alb ratio is associated with poor prognosis (*p*= 0.005) (Table 1). Compared with patients who had CRP/Alb ratio < 0.441, those with CRP/Alb ratio ≥ 0.441 had a 1.34 times higher chance of death (Table 3). By multivariate analysis, when adjusted for other variables, including cancer stage, CRP/Alb ratio independently predicted the overall survival of patients with SCLC (*p* = 0.025) (Table 3). Subgroup analysis suggested that OS in CRP/Alb ratio <0.441 group was significantly longer than those with CRP/Alb ratio ≥0.441 in limited stage (*p* = 0.023). To our knowledge, this is the first study to evaluate the prognostic value of CRP/Alb ratio in patients with lung cancer. Furthermore, it is the first study to indicate that CRP/Alb ratio can predict the overall survival in SCLC. Other than PS, the CRP/Alb ratio is more objectively determined, and would be a simple, optimal, and inexpensive prognostic indicator in patients with SCLC. Although first-line treatment with chemotherapy and radiotherapy provide much clinical benefit for patients with SCLC, they are associated with severe adverse reaction. These include myelosuppression, anorexia, and radiation pneumonitis, which may suppress the immune system and negatively affect the nutritional status of patients. Therefore, it is essential that therapeutic decisions should take into consideration the curative effects versus treatment-induced toxicities. In this study, we demonstrate that CRP/Alb ratio may serve as a screening method to choose the appropriate treatment for patients with SCLC.

There are several limitations to our study. This includes being a retrospective and single-center study, which may limit the prognostic value of the CRP/Alb ratio. To minimize selection bias, we enrolled consecutive patients between January 2006 and December 2011. We also observed that by ultivariate analysis, the prognostic value of CRP/Alb ratio significantly correlates with limited stage in patients with SCLC. This suggests that CRP/Alb ratio is a prognostic factor, though a large-scale prospective validation study is needed. In summary, this study demonstrated that a systemic inflammation-based marker, such as CRP/Alb ratio, is an independent predictor of overall survival for patients with SCLC. The CRP/Alb ratio could be used to better predict prognosis in patients with SCLC.

## Additional Information

**How to cite this article**: Zhou, T. *et al.* Ratio of C-Reactive Protein/Albumin is An Inflammatory Prognostic Score for Predicting Overall Survival of Patients with Small-cell Lung Cancer. *Sci. Rep.* **5**, 10481; doi: 10.1038/srep10481 (2015).

## Figures and Tables

**Table 1:** Basic characteristics of all patients and univariate survival analysis.

**Variables**	**Cases, *N***	**Proportion, %**	**Median OS, months (95% CI)**	***P* value^b^**
**Age, years**	59 (23-82)^a^			0.049

**Gender**				
Male	316	86.1	16.7(14.75-18.65)	0.077
Female	51	13.9	18.7(11.61-27.39)	

**Smoking**				
Smoker	302	82.3	16.8(14.69-18.91)	0.917
Never-smoker	65	17.7	16.0(12.61-19.33)	

**Disease stage**				
Limited stage	180	49.0	22.1(17.10-27.10)	<0.001
Extensive stage	187	51.0	14.2(11.61-16.72)	

**PS**				
0	195	53.1	17.0(15.01-19.06)	<0.001
1	142	38.7	18.7(16.14-21.20)	
2	30	8.2	9.3(7.94-10.73)	

**CRP/Alb**				
≥0.441	128	34.9	13.7(11.86-15.61)	0.005
<0.441	239	65.1	18.9(16.93-20.87)	

**LDH, U/L**				
**N**ormal range	220	59.9	19.2(17.52-20.89)	<0.001
Abnormally elevated	147	40.1	13.3(11.06-15.61)	
				
**Chemotherapy regimen**				
Etoposide-based	315	85.8	17.2(15.34-19.13)	0.685
Others	52	14.2	13.2(7.31-19.16)	
				
**Prophylacticcranial irradiation**				0.361
Yes	81	22.1	16.2(12.85-19.55)	
No	286	77.9	17.0(15.08-18.99)	

Note: ^a^Median (range); ^b^Log-rank test

Abbreviations: *OS*, overall survival; *CI*, confidence interval; *PS*, performance status; *CRP/Alb*, C-reactive protein/albumin; *LDH*, lactate dehydrogenase

**Table 2:** Clinicopathological characteristics of patients stratified by CRP/Alb ratio.

**Variables**	**CRP/Alb ratio ≥0.441, n (%)**	**CRP/Alb ratio <0.441, n (%)**	***P* value**
**Age( years)**			0.659
** <59**	108 (66.7)	54 (33.3)	
** ≥59**	131 (63.9)	74 (36.1)	
**Gender**			0.637
** **Male	204 (64.6)	112 (35.4)	
** **Female	35 (68.6)	16 (31.4)	
**Smoking**			0.669
** **smoker	195 (64.6)	107 (35.4)	
** **Never smoker	44 (67.7)	21 (32.3)	
**PS at diagnosis**			0.734
** **0	130 (66.7)	65 (33.3)	
** **1	91 (64.1)	51 (35.9)	
** **2	18 (60.0)	12 (40.0)	
**Stage**			0.584
** **Limited disease	120 (66.7)	60 (33.3)	
** **Extensive disease	119 (63.6)	68 (36.4)	
**LDH at diagnosis, U/L**			0.010
** **Normal range	155 (70.5)	65 (29.5)	
** **Abnormally elevated	84 (57.1)	63 (42.9)	
**Chemotherapy regimen**			0.432
** **Etoposide-based	208 (66.0)	107 (34.0)	
** **others	31 (59.6)	21 (40.4)	
**Prophylactic cranial irradiation**			0.793
** **Yes	185 (64.7)	101 (35.3)	
** **No	54 (66.7)	27 (33.3)	

Abbreviations: *PS*, performance status; *LDH*, lactate dehydrogenase; *CRP/*Alb, C-reactive protein / albumin.

**Table 3:** Results from Cox Regression Model (Adjusted for Age, Sex, Disease stage, PS and the CRP/Alb ratio).

**Variables**	**Hazard Ratio**	**95% CI**		***P*** **value**
		**LL**	**UL**	
**Age (per 10 years’ increment)**	1.26	0.98	1.62	0.073
				
**ECOG-PS**				
0	1.00	-	-	-
1	0.94	0.73	1.22	0.654
2	3.13	1.94	5.05	<0.001
				
**CRP/Alb ratio**				
≥0.442	1.00	-	-	-
<0.442	1.34	1.04	1.73	0.025
				
**Disease stage**				
Limited stage	1.00	-	-	-
Extensive stage	2.13	1.65	2.76	<0.001
				
**LDH (per 100U/L increment)**				
Normal range	1.00	-	-	-
Abnormally elevated	1.41	1.10	1.83	0.008

**Abbreviations:** CI, confidence interval; LL, lower limit; UL, upper limit; PS, performance status; CRP/Alb, C-reactive protein / albumin; LDH, lactate dehydrogenase.Ratio of C-Reactive Protein/Albumin is An Inflammatory Prognostic Score for Predicting Overall Survival of Patients with Small-cell Lung Cancer
